# Vaccination—A Review

**Published:** 1917-02

**Authors:** Theo. Y. Hull

**Affiliations:** San Antonio, Texas


### Vaccination—A Review.

BY DR. THEO. Y. HULL, SAN ANTONIO, TEXAS.

In view of the prevalence of smallpox at this time, it may not be amiss to review briefly some of the main facts concerning the disease.

Variola, pocken or smallpox, in historic importance, is said to outrank all other epidemic diseases. None other has pursued the human race with such persistence and malignity. It seems to have been indigenous in Hindustan from very ancient times. The old Brahman mythology recognized a special god for the disease. The Chinese records show that it was known in China over 3000 years ago, and it is still there. When it spread westward is not known.

The first noted epidemic in Western Asia occurred at Mecca about the middle of the sixth century. From thence it spread to Arabia, North Africa and Asia Minor. About the same time it appeared in Europe. A fatal epidemic broke out in Southern France and Northern Italy in 581 A. D. It later invaded England and Denmark and reached Iceland in 1241 A. D. Two hundred years later it destroyed the Norse Colony in Greenland. Before 1527 it reached Mexico with the Spanish conquerors and wrought havoc among the Aztecs.

It became practically universal during the eighteenth century. No land was free from the pestilence for any length of time. It outclassed the bubonic plague. It became the dominant pandemic.

During the eighteenth century it reached its greatest intensity. The mortality was said to be frightful. In England, smallpox was responsible for 10 per cent of the total mortality. In France, 30,000 died annually. In Prussia, 26,646 died from the disease during 1796. Even those who escaped death were disfigured for life. The great prevalence of the disease gave rise to the expression, "From smallpox and love but few escape"—the humor of despair.

Although physicians were able to render little aid in treating the disease, they learned that it was specific and contagious and began employing prophylactic measures, such as isolation and fumigation. About the same time another fact was discovered: That is, that one attack usually rendered the individual free from future attacks. These discoveries led the way to future advances in prophylaxis. It was also observed that persons who had suffered only the lightest attacks were as free from future attacks as those who had had the more severe forms. This led to an attempt to acquire smallpox under the most favorable conditions.

In some regions the custom of "smallpox purchase" prevailed. Healthy persons were exposed for a brief time to mild cases of the disease in the hope that mild cases would occur in the exposed individuals, but the "purchaser" thus exposed did not always get what he bargained for—a mild case.

In 1721 the information was brought to England through Constantinople that in the far East a certain form of inoculation had been practiced from ancient times. This was the direct inoculation of the smallpox virus from one individual to another. The guiding idea was the same as in the "Pockenkaufer" that the virus from a mild case inoculated into a healthy person would produce in the inoculated a mild case, which would produce lasting immunity. This, likewise, was found not free from danger, but it laid the foundation for future attempts to produce immunity without danger to the inoculated.

This procedure was brought to England by the wife of the English ambassador at Constantinople. Her six-year-old son was inoculated by a Greek physician in 1717. Later, on her return to England (1721), her daughter was likewise inoculated. This was the beginning of a practice carried out in Europe during the eighteenth century. These inoculations, carefully and conscientiously carried out, usually resulted favorably, but were not found entirely free from bad results. But carelessness, ignorance and charlatanism soon entered into the equation and, in consequence of the bad results following, the regular profession and the clergy protested against it.

In continental Europe the first period of inoculation was of brief duration. A great controversy arose over the subject, in which the names of De Haen, De la Condomin, Tissot, Hensler and Gatte appear. The violent opposition of De Haen reacted in favor of the procedure.

The second period of inoculation followed Gatte's teachings (1760) and lasted until 1798. But in spite of improved technique, severe cases were caused by such inoculation, and fatal cases still occurred. Gregory places the fatality of inoculation as practice during the last half of the eighteenth century at 1 to 300.

Another objection raised was that inoculated smallpox acted exactly like natural smallpox, and when not carefully guarded favored the spread of the disease and the occurrence of epidemics.

The eighteenth century closed with the rapid decline of the inoculation of susceptible persons with human virus. The nineteenth century opens with the extended experiments upon animals. In 1807, Gassner inoculated children with virus obtained from cows which had been inoculated with human virus from active cases of smallpox. Thiele inoculated cows with human virus, using the udder as the site of inoculation. With the virus obtained from the pustules formed on the udder, children were inoculated. Thiele inoculated over 3000 persons in this manner. Ceely inoculated over 2000 persons in the same way. Both proved that the attenuated virus from the cow afforded protection from smallpox.

Several animals are subject to pock-like diseases. As this disease—cowpox—appears in cattle, it is of the most importance to us, on account of its relation to vaccination. When cowpox appears spontaneously among cattle, it is designated as original or natural vaccinia. This original vaccinia is the foundation of modern vaccination. It was noticed that natural vaccinia attacked a cow but once in a lifetime. It was also observed that the disease was easily transferred from the cow to human beings. Milkmaids were the ones most frequently attacked by it. It was further observed that individuals who had suffered an attack of cowpox or vaccinia were immune to subsequent attacks of smallpox. This fact formed the starting point for the theory of vaccination. Thiele and Ceely and many others believed that vaccinia, as it appears in cows, was only a modified smallpox.

Up to this time the attempt to immunize human beings against smallpox had passed through three stages: (1) The inoculation of healthy individuals with human virus taken from mild cases of actual smallpox. (2) The inoculation of individuals, especially children, with virus obtained from animals inoculated with human virus from actual cases of smallpox. (3)

The inoculation of healthy individuals with virus obtained from active cases of cowpox.

It may be said that vaccination did not spring like Pallas Athene from the brain of any one person, but was a natural evolution. To Edward Jenner, of England, belongs the credit of having demonstrated to the medical profession both the possibility of immunization against smallpox and its harmlessness. His name is justly recorded among the benefactors of the human race. But long before his day it had been observed that persons who had not suffered an attack of smallpox, but had been infected with cowpox, remained immune though subsequently exposed.

In Mexico, in certain parts of Asia and in some parts of Europe the belief was prevalent that an attack of cowpox protected the individual from smallpox. Some experimental work was done by Sutton and Fenster and some actual vaccinations were performed by the laity with the distinct idea of producing immunity before the time of Jenner. Jenner in his experiments proceeded along definite lines. He first attempted to inoculate with human smallpox virus persons who had at some previous time been accidentally infected with cowpox. There were sixteen cases in all. None of these developed smallpox, though some of them had suffered from the cowpox infection many years before. He next inoculated an individual with virus obtained from a case of accidentally acquired cowpox. Typical cowpox developed. The patient was subsequently inoculated with smallpox virus with negative results. Jenner thus proved that humanized cowpox or vaccinia could be transmitted and that such transmission protecteed individuals from smallpox infection.

He now repeated his experiments with humanized cowpox virus up to the fifth generation with uniformly successful results. Jenner made the first report of his experiments to the medical profession in 1798, a second report in 1799 and a third in 1800. His work was tested and confirmed by the most noted physicians of Europe. In the earlier years of the new century Jenner's method of vaccination had the active support of the entire profession of Europe.

A marked fall in the mortality of the disease followed whereever vaccination was thoroughly carried out. The result was clearly seen during the first decade, during the second it was still more marked and the third showed the effects were permanent.

In 1806, Bavaria enacted a vaccination law. This law required that all children, without exception, be vaccinated during the first year, and among other things made vaccination compulsory during an epidemic of smallpox. A number of governments followed Bavaria's lead. Sweden required universal vaccination before entrance into the empirical schools. In Italy, vaccination met with great favor. Sacco became Jenner's most ardent disciple. Vaccination became a family custom. While England was the cradle of vaccination, Italy became the cradle of pure animal vaccination. The production of an unobjectionable vaccine, a pure bovine vaccine from a case of original cowpow which was artificially transmitted from cow to cow, was the work of the Italians.

It is not to be supposed that so great a revolution in medicine as vaccination could be brought about without opposition. Such opposition usually thrives on the failure of any reform to measure up to the utmost claims of the most enthusiastic supporters. As it began to be observed that persons who had once been successfully vaccinated later contracted smallpox and the number increased with each decade, faith in vaccination became shaken and reaction set in. In the bitter controversy that followed the idea of revaccination was born. It generally became clear that a single vaccination could not be relied upon for permanent protection.

With the success of vaccination also spread the anti-vaccination agitation, which rejected the whole procedure as injurious to the common good. This agitation was increased by the discovery that syphilis occasionally followed vaccination. The controversy went on for twenty years, in which vaccination was accused of causing the spread not only of syphilis, but scrofula and other constitutional diseases.

To end this discussion, the English General Board of Health in 1855 prepared four questions which it submitted to 542 medical authorities in Europe, Asia and America. These questions asked:

1. Whether successful vaccination conferred protection against smallpox in most cases.

2. Whether the lessened susceptibility of vaccinated individuals to smallpox predisposed them to other infectious diseases.

3. Whether syphilis, scrofula and other constitutional diseases could be transmitted through vaccination.

4. Whether the general vaccination of children was to be recommended.

To these questions, 542 replies were received during the next two years. These replies are to be found in the English Blue Book of Vaccination, 1857.

Of these replies from medical authorities, 540 answered the first question in the affirmative. The answer to the second was a universal negative. There was some difference of opinion in reference to the third. The fourth was answered by 540 in the affirmative.

The revaccination movement began in 1824. This procedure was recognized officially by several countries in the next few years. By 1850, revaccination was compulsory in the German army. In 1874, the Imperial Vaccination Law of Germany was passed. This law required: (1) That every child must be vaccinated during the first year of its life. (2) That every pupil in the public or private schools must be revaccinated during the twelfth year. It is interesting to know that this law permitted the use of human vaccine lymph. Later, 1885, a pure animal lymph was adopted for vaccination and revaccination.

While the primary vaccination done in infancy is generally considered to protect the individual for ten years and a revaccination to possess the same value, it has become a general practice to revaccinate school children every three, five or seven years.

It is worthy of note, wherever vaccination has been neglected, epidemics of smallpox have broken out.

Vaccination and revaccination, like every other protective measure, should be judged by the results obtained in decreasing the morbidity and in lowering the death rate among those who may become infected.

Vaccination was introduced in 1798, and a careful study of its effectiveness warrants the conclusion that it has not only greatly reduced the frequency of smallpox, but also reduced its fatality. During the eighteenth century good authorities state that 95 per cent of the inhabitants of Europe suffered from smallpox. During the nineteenth century in Central Europe the morbidity decreased to 5 per cent. As to mortality during the eighteenth century, it stood next to tuberculosis as a cause of death, 8 per cent of all deaths being due to the disease. In the nineteenth century, after the introduction of vaccination, the mortality amounted to less than 1 per cent.

No better evidence of the beneficial results of vaccination can be found anywhere than in Sweden, for nowhere else are more accurate records of the mortality of smallpox, extending over so long a period, to be found. They cover a period of 112 years —1744 to 1855. Vaccination was first performed in Sweden in 1801. It came into general use in 1810. It was made a legal institution in 1816. The record extends an aqual distance on both sides of the beginning of vaccination.

This time is divided into three periods: (1) The prevaccination period, (2) the transition period, and (3) the vaccination period. During the prevaccination period—1744 to 1801—among every million inhabitants 2050 deaths from smallpox occurred. During the transition period—1801 to 1810—the mortality was reduced to 686 per 1,000,000 population. During the vaccination period—1810 to 1855—the mortality dropped to 169 per 1,000,000. In other words, according to this showing, a city of the size of San Antonio during the prevaccination period would have over 200 deaths annually from smallpox, while during the vaccination period it would have had 17 deaths from the same cause. In fact, during the five-year period—1910 to 1914—there were 6 deaths from smallpox.

The Medical Faculty of the University of Prague (1856) reported that in Bohemia among a population of 3,039,722 there were 7663 deaths annually from smallpox during the seven years preceding the introduction of vaccination—1796 to 1802. During the twenty-four-year period—1832 to 1855—of compulsory vaccination among a population of 4,248,155 there died annually of smallpox 287. During the first period the mortality from smallpox was to the general mortality as 1 to 12, while in the latter period it was 1 to 458.

The first effect of vaccination is thus clearly seen. A second effect may be seen in the fact that, in a mixed population of vaccinated and non-vaccinated, the vaccinated were attacked by the disease comparatively less often and more mildly, and with a much smaller relative mortality than were the non-vaccinated.

During the epidemic of 1871 in Leipzig 688 cases of smallpox were treated in the polyclinic, 417 vaccinated and 271 non-vaccinated. The mortality among the vaccinated was 18 or 4.3 per cent, while among the non-vaccinated there were 114 deaths or 42.1 per cent, a ratio of 1 to 10.

Chemnitz, Saxony, in 1870-71 had a population of 64,222, vaccinated 53,891, non-vaccinated 5712 and protected by previous smallpox 4652. During the epidemic of 1870-71, 3596 cases occurred or 5.16 per cent of the entire population. The relative morbidity among the vaccinated and the non-vaccinated was as 1 to 26, while the relative mortality was 1 to 326. In other words, among 53,891 vaccinated, only 7 died. Among the 5712 non-vaccinated 242 died and among the 4652 protected by previous attack of smallpox no cases occurred. The ratio was 1 to 13 in favor of vaccination.

After revaccination became an established procedure, both the morbidity and the mortality of smallpox were further reduced. The German vaccination law requiring revaccination every twelve years went into effect in 1875.

In Prussia—1816 to 1870—the mortality from smallpox varied from 7.32 to 62.0 per 100,000 inhabitant annually. From 1875 to 1886, twelve years, the average mortality was 1.91 per 100,000. These figures scarcely need comment.

One of the chief arguments against vaccination was the alleged danger of transmitting syphilis. In the early days of vaccination this was a valid objection. Vaccine material taken from a syphilitic for the purpose of vaccinating a non-syphilitic is capable of transferring syphilis.

Today, with modern methods of procedure and modern methods of preparing the vaccine, no such objection can be valid.

On the whole, it may be said that vaccination is one of the greatest blessings ever bestowed upon the human race. One hundred years ago smallpox was a universal plague; today many physicians practice a lifetime without ever seeing a case.

---

Do you believe in national preparedness and then fail to keep yourself physically fit?

---

Dr. H. P. Hill is the Superintendent of the Robert Green Memorial Hospital, which has recently been opened in San Antonio. Eleven thousand dollars has been donated by Mr. Alexander Joske for the equipping of operating, clinical and X-ray rooms.

---

At a meeting of the North Texas Medical Association, which was held in Dallas on December 12th and 13th the following officers were elected: President Dr. Will Cantrell, Dallas; Vice-President, Dr. C. O. Harper, Fort Worth (re-elected); Secretary, Dr. H. L. Moore (re-elected), and Treasurer, Dr. D. L. Bettison, Dallas.

